# Management of Hyperthyroidism in Pregnancy: Comparison of Recommendations of American Thyroid Association and Endocrine Society

**DOI:** 10.1155/2013/878467

**Published:** 2013-05-22

**Authors:** Shahram Alamdari, Fereidoun Azizi, Hossein Delshad, Farzaneh Sarvghadi, Atieh Amouzegar, Ladan Mehran

**Affiliations:** Endocrine Research Center, Research Institute for Endocrine Sciences, Shahid Beheshti University of Medical Sciences, Tehran 19395-4763, Iran

## Abstract

Appropriate diagnosis and treatment of hyperthyroidism during pregnancy are of outmost importance, because hyperthyroidism has major adverse impact on both mother and fetus. Since data on the management of thyroid dysfunction during pregnancy is rapidly evolving, two guidelines have been developed by the American Thyroid Association and the Endocrine society in the last 2 years. We compare here the recommendations of these two guidelines regarding management of hyperthyroidism during pregnancy. The comparison reveals no disagreement or controversy on the various aspects of diagnosis and treatment of hyperthyroidism during pregnancy between the two guidelines. Propylthiouracil has been considered as the first-line drug for treatment of hyperthyroidism in the first trimester of pregnancy. In the second trimester, consideration should be given to switching to methimazole for the rest of pregnancy. Methimazole is also the drug of choice in lactating hyperthyroid women.

## 1. Introduction

Diagnosis of hyperthyroidism which occurs in 0.05 to 3.0% of pregnancies may be difficult in these women, as the symptoms and signs of nervousness, sweating, dyspnea, tachycardia, and cardiac systolic murmur are seen in most normal pregnancies as well [1, 2]. More specific findings of hyperthyroidism such as weight loss and goiter and/or autoimmune thyrotoxicosis like ophthalmopathy may suggest Graves' hyperthyroidism [3, 4]. In addition, the occurrence of transient hyperthyroidism of hyperemesis gravidarum or gestational thyrotoxicosis may complicate diagnosis in the first half of pregnancy [5]. The diagnosis of hyperthyroidism should always be confirmed by the measurement of circulating free T_4_ and TSH. However, serum T_4_ concentration (both total and free) varies during normal pregnancy. Optimal methods such as liquid chromatography and tandem mass spectrometry used to assess serum free T_4_ are expensive and not commonly always available [6]. Therefore estimation of FT_4_ index may be employed, but international reference ranges have not been available until recently and only one manuscript is under publication [7]. The normal reference range for TSH concentrations during each trimester of pregnancy must be developed by each laboratory [6, 8]. 

 Hyperthyroidism has well-documented adverse impacts on both mother and fetus [9]. Therefore recognition and proper management of hyperthyroidism during pregnancy are of outmost importance. Antithyroid medications are the mainstay of treatment of thyrotoxicosis during pregnancy, as thyroidectomy requires pretreatment with antithyroid drugs and may be complicated by the adverse effects of surgery and radioiodine therapy is contraindicated [1–4].

The rapidly evolving data on the management of thyroid disorders during pregnancy have been the impetus for development of many guidelines during the past few years. It is noteworthy that two guidelines on thyroid and pregnancy were documented in October 2011 [10] and August 2012 [11] by American Thyroid Association and Endocrine Society, respectively. It is the aim of this paper to compare the recommendations of these two guidelines regarding management of hyperthyroidism during pregnancy. 

## 2. Methods

 The section of thyrotoxicosis in pregnancy, pages 1093–1096 of the Guidelines of the American Thyroid Association [10] and the section on the management of hyperthyroidism: Maternal and fetus aspects, pages 2550–2553 of the Endocrine Society Clinical Practice guidelines [11] were reviewed and their recommendations for each topic were compared; the ones that were more complete were selected and are included in tables. If a recommendation of the other organization was different or more informative, it was cited, otherwise the word “same” was used. If similar information regarding a recommendation of one organization was present in the recommendation or text of guidelines of the other organization, words (R) or (T) were added, respectively.

## 3. Results

 The ATA Guidelines posed 14 questions and responses were gathered to 14 recommendations [10]. In the Endocrine Society Clinical Practice Guidelines, a total of 13 recommendations were given for the management of hyperthyroidism in pregnancy, including 5 recommendations for management of maternal aspects of hyperthyroidism, 5 for management of fetal aspects, and 3 for gestational hyperemesis and hyperthyroidism [11]. Comparison of the recommendations of both organizations on the management of hyperthyroidism before pregnancy and on the diagnosis of hyperthyroidism during pregnancy is summarized in Table 1. The importance of low TSH in the first trimester, doubtful value of ultrasound in differentiating cause of hyperthyroidism and contraindication of radioiodine uptake and scanning have been included in both recommendations. Regarding differentiation of Graves' disease and gestational thyrotoxicosis, TSH receptor antibodies (TRAb) determination had been recommended.

Table 2 compares recommendations of ATA and Endocrine Society on the management of hyperthyroidism during pregnancy. The use of propylthiouracil (PTU) has been recommended by both organizations during the first trimester of pregnancy, followed by methimazole (MMI) after the first trimester. Endocrine society guideline states that MMI may be prescribed if PTU is not available or if a patient cannot tolerate or has an adverse response to PTU and it also recommends that practitioners should use their clinical judgment in switching patients from one drug to another. Combinations of regiment of T4 with antithyroid drugs have not been recommended and an indication of surgery has been described by both guidelines. Only Endocrine Society guideline recommends liver function tests in pregnant women on PTU every 3-4 weeks.

Comparison of two recommendations on fetal aspects of hyperthyroidism in pregnancy is almost similar (Table 3), stressing the importance of measurement of TRAb at 20–24 weeks of gestation, consulation with and expert obstetrician, and following up of fetal thyroid dysfunction.

Table 4 compares the recommendations of both organizations on the management of gestational hyperthyroidism. They recommend supportive therapy and avoidance of antithyroid therapy. Both guidelines state that subclinical hyperthyroidism during pregnancy does not require any treatment.

## 4. Discussion

Graves' disease is the most common cause of autoimmune hyperthyroidism in pregnancy. It has been reported in about 0.5% of pregnancies. It may be the first manifestation of the disease or may present as a recurrent episode in a woman with past history of hyperthyroidism, or a pregnancy in a women on antithyroid drugs [12]. Other rare causes of hyperthyroidism included MNG, toxic adenoma, and factitious hyperthyroidism. More frequent than Graves' disease as the cause of hyperthyroidism is the syndrome of gestational hyperthyroidism or gestational transient thyrotoxicosis, diagnosed in about 3–5% of pregnancies and includes women with hyperemesis gravidarum, multiple pregnancies, and hydatidiform mole [13].

Like other autoimmune diseases, the activity of Graves' disease is exacerbated during the first trimester of gestation and decreased during the latter half of pregnancy, to be exacerbated again shortly after delivery or late in the postpartum period [1, 14, 15].

Many of symptoms of hyperthyroidism such as tachycardia, tremor, warm and moist skin, and systolic murmur may be present in normal pregnancy. In the first trimester of gestation, the normal elevation in TT4 and TT3, due to estrogen-induced increase in TBG concentration, and hCG thyroid stimulation with suppression of serum TSH, may pause difficulties in the diagnosis of maternal hyperthyroidism [16].

Management of hyperthyroidism during pregnancy requires special considerations because maternal thyroid disease could have adverse effects on the mother, fetus, and neonate. 

Poorly treated or untreated maternal overt hyperthyroidism may affect pregnancy outcome, mainly as a result of complications such as preeclampsia, preterm delivery, intrauterine growth restriction, low birth weight, fetal hydrops, and stillbirths [17–19]. Other complications include congestive heart failure, thyroid storm, and postpartum bleeding. Inappropriate doses of antithyroid drugs and their crossing from the placenta could be the cause of fetal hypothyroidism with or without fetal goiter. [20]. Frequent determination of maternal thyroid hormones, with the target to keep the serum FT_4_ or TT_4_ in the 1/3 upper limit of reference range, is the most effective way to prevent such complications [1, 21]. In addition, the transplacental passage of maternal TRAb may cause fetal or neonatal Graves' disease [22]. If the mother remains hyperthyroid during most of the pregnancy, high maternal thyroxine values, crossing the placenta may produce neonatal central hypothyroidism [23].

Postpartum thyrotoxicosis due to Graves' disease may be treated with radioiodine but it requires radiation safety measurements for infant and is contraindicated if the mother is breast-feeding. Antithyroid drugs are the mainstay of the treatment of postpartum thyrotoxicosis due to Graves' disease [24]. Recent investigations conclude that neither PTU up to 300 mg nor MMI up to 30 mg daily cause any alterations in thyroid function and physical and mental development of infants breast-fed by lactating thyrotoxic mothers [25–28]. However, MMI is the preferred drug, because of the potential necrosis of the liver in either mother or child. 

 Management of hyperthyroidism during pregnancy and lactation requires special considerations and should be meticulously implemented to provide best care to pregnant woman and prevent any adverse effects on the mother, fetus, or neonate. A comparison of recommendations of the American Thyroid Association (2011) and the Endocrine Society (2012) on various aspects of diagnosis and treatment of hyperthyroidism in pregnancy does not reveal any disagreement or controversy. Almost all the information given by one organization could be found in the text or recommendations of the other. Although the authors of both guidelines should be applauded for their ability to synthesize complex data into high quality guidelines, the presence of two guidelines in a time distance of only 10 months by 2 prestigious organizations may perplex clinicians to select one guideline or the other for management of their patients. In the opinion of the authors of this paper either of the two guidelines may be used for appropriate and up-to-date management of thyrotoxic pregnant women.

## Figures and Tables

**Table 1:** Comparison of recommendations of American Thyroid Association and Endocrine Society on the management of hyperthyroidism before pregnancy and on the diagnosis of hyperthyroidism and pregnancy.

Topic	Recommendations	
	American Thyroid Association (2011)	Endocrine Society (2012)
Management before pregnancy	Same (R and T)	For overt hyperthyroidism due to Graves' disease or thyroid nodules, antithyroid drug (ATD) therapy should be either initiated (before pregnancy if possible, and for those with new diagnoses) or adjusted (for those with a prior history) to maintain the maternal thyroid hormone levels for free T4 at or just above the upper limit of the nonpregnant reference range, or to maintain total T4 at 1.5 times the upper limit of the normal reference range or the free T4 index in the upper limit of the normal reference range.

Thyroid function tests	In the presence of a suppressed serum TSH in the first trimester (TSH < 0.1 mIU/L), a history and physical examination are indicated. FT4 measurements should be obtained in all patients. Measurement of TT3 and TRAb may be helpful in establishing a diagnosis of hyperthyroidism.	Same (R)

Ultrasonography	There is not enough evidence to recommend for or against the use of thyroid ultrasound in differentiating the cause of hyperthyroidism in pregnancy.	None

Scanning and uptake	Radioactive iodine (RAI) scanning or radioiodine uptake determination should not be performed in pregnancy.	None

Differentiation of Graves disease and gestational thyrotoxicosis	Same (T)	Differentiation of Graves' from gestational thyrotoxicosis is supported by the presence of clinical evidence of autoimmunity, typical goiter, and presence of TSH receptor antibodies (TRAb). TPO-Ab may be present in either case.

**Table 2:** Comparison of recommendations of American Thyroid Association and Endocrine Society on the treatment of hyperthyroidism in pregnancy.

Topic	Recommendations	
	American Thyroid Association (2011)	Endocrine Society (2012)
Antithyroid (ATD) treatment	PTU is preferred for the treatment of hyperthyroidism in the first trimester, and patients on MMI should be switched to PTU if pregnancy is confirmed in the first trimester. Following the first trimester, consideration should be given to switching to MMI.	Propylthiouracil (PTU), if available, is recommended as the first-line drug for treatment of hyperthyroidism during the first trimester of pregnancy because of the possible association of methimazole (MMI) with specific congenital abnormalities that occur during first trimester organogenesis, and MMI may also be prescribed if PTU is not available or if a patient cannot tolerate or has an adverse response to PTU. Practitioners should use their clinical judgment in choosing the ATD therapy, including the potential difficulties involved in switching patients from one drug to another. If switching from PTU to MMI, thyroid function should be assessed after 2 weeks and then at 2- to 4-week intervals.

Combination of LT4 and ATD	A combination regimen of T4 and an ATD should not be used in pregnancy, except in the rare situation of fetal hyperthyroidism.	None

Monitoring liver function in women on PTU	None	Although liver toxicity may appear abruptly, it is reasonable to monitor liver function in pregnant women on PTU every 3-4 weeks and to encourage patients to promptly report any new symptoms.

Monitoring of thyroid function	In women being treated with ATDs in pregnancy, FT4 and TSH should be monitored approximately every 2–6 weeks. The primary goal is a serum FT4 at or moderately above the normal reference range.	Same (T)

Surgery	Same (R and T)	Subtotal thyroidectomy may be indicated during pregnancy as therapy for maternal Graves' disease if (1) a patient has a severe adverse reaction to ATD therapy (2) persistently high doses of ATD are required (over 30 mg/d of MMI or 450 mg/d of PTU) or (3) a patient is nonadherent to ATD therapy and has uncontrolled hyperthyroidism. The optimal timing of surgery is in the second trimester.

**Table 3:** Comparison of recommendations of American Thyroid Association and Endocrine Society on the fetal aspects of hyperthyroidism in pregnancy.

Topic	Recommendations	
	American Thyroid Association (2011)	Endocrine Society (2012)
Thyroid receptor antibodies (TRAb)	If the patient has a past or present history of Graves' disease, a maternal serum determination of TRAb should be obtained at 20–24 weeks gestation.	TRAb should be measured by 22-week gestational age in mothers with (1) current Graves' disease; or (2) a history of Graves' disease and treatment with 131I or thyroidectomy before pregnancy; (3) a previous neonate with Graves' disease; or (4) previously elevated TRAb

Fetal Surveillance	Fetal surveillance with serial ultrasounds should be performed in women who have uncontrolled hyperthyroidism and/or women with high TRAb levels (greater than three times the upper limit of normal). A consultation with an experienced obstetrician or maternal-fetal medicine specialist is optimal. Such monitoring may include ultrasound for heart rate, growth, amniotic fluid volume, and fetal goiter.	In women with TRAb or thyroid-stimulating Ig elevated at least 2- to 3-fold the normal level and in women treated with ATD, maternal free T4, and fetal thyroid dysfunction should be screened for during the fetal anatomy ultrasound done in the 18th–22nd week and repeated every 4–6 weeks or as clinically indicated. Evidence of fetal thyroid dysfunction could include thyroid enlargement, growth restriction, hydrops, presence of goiter, advanced bone age, tachycardia, or cardiac failure, if fetal hyperthyroidism is diagnosed and thought to endanger the pregnancy, treatment using MMI or PTU should be given with frequent clinical, laboratory, and ultrasound monitoring.

Umbilical blood sampling	Cordocentesis should be used in extremely rare circumstances and performed in an appropriate setting. It may occasionally be of use when fetal goiter is detected in women taking ATDs to help determine whether the fetus is hyperthyroid or hypothyroid.	Same (R)

Evaluation of newborn	Same (T)	All newborns of mothers with Graves' disease (except those with negative TRAb and not requiring ATD) should be evaluated by a medical care provider for thyroid dysfunction and treated if necessary.

**Table 4:** Comparison of recommendations of American Thyroid Association and Endocrine Society on other aspects of hyperthyroidism in pregnancy.

Topic	Recommendations	
	American Thyroid Association (2011)	Endocrine Society (2012)
Management of gestational hyperthyroidism	The appropriate management of women with gestational hyperthyroidism and hyperemesis gravidarum includes supportive therapy, management of dehydration, and hospitalization if needed. ATDs are not recommended for the management of gestational hyperthyroidism. Same (T)	Most women with hyperemesis gravidarum, clinical hyperthyroidism, suppressed TSH, and elevated free T4 do not require ATD treatment. Clinical judgment should be followed in women who appear significantly thyrotoxic or who have in addition serum total T3 values above the reference range for pregnancy. Beta blockers such as metoprolol may be helpful and may be used with obstetrical agreement. Women with hyperemesis gravidarum and diagnosed to have Graves' hyperthyroidism (free T4 above the reference range or total T4 > 150% of top normal pregnancy value, TSH < 0.01 mIU/liter, and presence of TRAb) will require ATD treatment, as clinically necessary.

Subclinical hypothyroidism	Same (T)	There is no evidence that treatment of subclinical hyperthyroidism improves pregnancy outcome, and treatment could potentially adversely affect fetal outcome.
